# Application of Nanocomposites from Bees Products and Nano-Selenium in Edible Coating for Catfish Fillets Biopreservation

**DOI:** 10.3390/polym14122378

**Published:** 2022-06-12

**Authors:** Dareen M. Youssef, Fawzia A. Alshubaily, Ahmed A. Tayel, Mousa A. Alghuthaymi, Mahmoud A. Al-Saman

**Affiliations:** 1 Department of Fish Processing and Biotechnology, Faculty of Aquatic and Fisheries Sciences, Kafrelsheikh University, Kafrelsheikh 33516, Egypt; dareenmahmoud30@gmail.com; 2 Biochemistry Department, Faculty of Science, King Abdulaziz University, Jeddah 21589, Saudi Arabia; falshibli@kau.edu.sa; 3 Department of Biology, Science and Humanities College, Shaqra University, Alquwayiyah 11726, Saudi Arabia; 4 Department of Industrial Biotechnology, Genetic Engineering and Biotechnology Research Institute, University of Sadat City, El-Sadat City 32897, Egypt; mahmoud.alsaman@gebri.usc.edu.eg

**Keywords:** antimicrobial, biosynthesis, chitosan, nanomaterials, propolis, seafood quality

## Abstract

Bee products, e.g., chitosan and propolis (Pro), have extraordinary importance in many disciplines including food biopreservation. Fish meat is highly susceptible to vast spoilage, especially catfish (*Clarias gariepinus*) products. The current work involved the extraction of bees’ chitosan nanoparticles (BCht), Pro, Pro-mediated SeNPs and their composites, to evaluate them as potential antimicrobial and preservative nano-compounds, for the preservation of catfish fillets and augment their quality. BCht was extracted from bees (*Apis mellifera*) corpses and had a 151.9 nm mean particle diameter. The Pro was used for biosynthesis of SeNPs, which had 11.2 nm mean diameters. The entire compounds/composites exhibited powerful antibacterial acts against *Escherichia coli*, *Staphylococcus aureus* and *Salmonella typhimurium*, where *S aureus* had the uppermost resistance. BCht/Pro/SeNPs were the most forceful toward all bacterial strains. The constructed edible coatings (ECs) from produced compounds/composites (BCht, Pro, Pro/SeNPs, Pro/BCht and BCht/Pro/SeNPs) had elevated efficiency for preserving catfish fillets during cold storages for 7 days. The microbiological (total counts, psychrophilic bacteria, yeast and molds), spoilage chemical parameters (TVB-N, TBARS) and sensorial attributes (appearance, odor, color, overall quality) of ECs-treated fillets indicated the nanocomposite’s efficiency for protecting the fish from microbial growth, the progress of chemical spoilage indicators and maintaining the sensorial quality of treated stored fillets. The most effective nanocomposite for maintaining the entire fillet’s quality was the BCht/Pro/SeNP. The based ECs on BNCt, Pro/SeNPs and their nanocomposites could be endorsed for prospective employment in the biopreservation of various seafoods.

## 1. Introduction

Chitosans are de-acetylated biopolymers that are derived principally from chitin and possess numerous bioactivities and applications as human-friendly molecules [1]. Chitosans are extraordinary agents for employment in nutrition, pharmaceutics, biomedicine, therapeutic, antimicrobial, anticancer, food preservation and drug-carrying branches [1,2,3,4]. The entire bioactivities of chitosan are influentially augmented via its transformation to “nano” forms due to the significant increment of particles’ surfaces and reactivity [5,6]. The diverse chitosan bases include crustacean waste (the accustomed source), fungi biomass or insect skeletons [3,7,8]. Honeybee (*Apis mellifera* L.) is an insect that could provide humans with numerous valuable products. In addition to honey (the main products of bees), many other products with high values could be attained, including propolis, venom and chitosan [9]. As the bee’s cuticle is comprised of 30–50% chitin, and because beehives are renewed by 60–80% each year, which could provide 6–10 million kilograms of bee corpora, honeybees could be suggested as a promising source for chitosan extraction [7,9,10,11].

Propolis (bee glue) consists of natural resinous materials which are collected by bee workers from various plants’ exudates and buds and then intermingled with pollen, wax and bee enzymes; the source and structure of propolis constituents could influence its pharmacological and therapeutic bioactivities [12]. Bees habitually use propolis to seal their hives against other biological invaders, e.g., insects and microorganisms; thus, propolis substances act as potent natural antibiotics to combat bacterial, fungal or viral infections [13]. The principal bioactivities and health-promoting attributes of propolis included its usages as antibacterial, anthelmintic, antiulcer, antiviral, antifungal, antioxidant, hepatoprotective, antiradiation, antimutagenic, antitumor, cyto- and chemopreventive, anti-angiogenic, wound healing, anti-inflammatory, immune-modulating, anti-diabetic, cardioprotective, cicatrizing, local anesthetic, and food biopreservative agents [14,15].

More than 850 components have been identified in different propolis types around the world. These phytochemical groups include: volatile oils, alcohols, flavonoids, alkanes, aromatic acids, terpenoids, sugar alcohols, aldehydes, chalcones, amino acids, fatty acids, vitamins, sugars and hydrocarbons, wax esters, ketones, phenols, glycerol derivatives and trace minerals [13,15,16]. However, the propolis extract (Pro) exhibited high potentiality for reducing metal ions to their nanoforms, due to its remarkable reducing, capping and stabilizing activities [12,17].

The development of biocompatible nanoparticles (NPs) has been the topic of discussion worldwide owing to the NP’s unique chemical, physical, and biological physiognomies [18]. For overcoming the potential limitations of NP synthesis (such as the release of toxic/hazardous chemicals and elevated energy expenditure, from the usages of conventional methods), biological approaches for NP synthesis (green or bio-synthesis) were proved as effective alternatives to provide prominent NPs with augmented structural, catalytic and electromagnetic properties, the least toxicity and cost-effectiveness [18,19,20,21].

Selenium (Se) is an essential micronutrient for human health due to its antioxidative and co-enzymatic roles in the body; a Se deficiency can frequently lead to hypothyroidism, heart disease, and immune system weakening [22,23]. The synthesized nanoparticles from Se (e.g., SeNPs) have an outstanding bioefficacy, minimal toxicity and higher biocompatibility when compared with other forms of organic/inorganic Se compounds, especially with biosynthesis application [22,23,24,25,26]. The Se toxicity could appear with human adult consumption of ≥3200 μg/day [22,23]. The excellent nutraceuticals and therapeutic aptitudes of biosynthesized SeNPs were prominent among other metals NPs, including their antimicrobial, antioxidant, antifungal, anticancer and immune-stimulation properties [21,24,27]. Although many reports stated that SeNP biosynthesis uses numerous plant derivatives [19,20,27,28,29,30], including essential oils, leaves, fruits and bark extracts; only limited investigations studied the Pro usages of SeNPs [12].

Fish meat is the foremost and most economical source of animal proteins; its value and importance increased with appropriate processing and preserving approaches [28]. From them, the flesh of African catfish (*Clarias gariepinus*) has a comparably elevated protein content (~16.9–17.9%) and low fat percentage (~3.95–7.57%); they were positioned as the ideal species for further utilization because of their endurance in diverse conditions and their extraordinarily high growth rate and processing ability [29,30]. However, harvested catfish are frequently subjected to numerous pathogenic microorganisms and spoilage conditions, which intensify public concerns regarding their products and require additional verifications of their potential usages and safety [30].

The approaches used for fresh seafood preservation and the elimination of pathogenic/spoilage parameters involve the reduction of storage temperature (e.g., cooling, freezing) and the use of food-grade preservatives with antimicrobial and antioxidant potentialities [2,29,31]. The composition of multiple biopreservative agents (e.g., biopolymers, plant extracts and nanomaterials) exhibited additional powers for protecting foodstuffs from spoilage conditions and attack by pathogenic microbes [31,32,33].

This current study, accordingly, targeted the extraction of bees’ chitosan nanoparticles (BCht), Pro, Pro-mediated SeNPs and their composites, to evaluate them as potential antimicrobial and preservative nano-compounds for the preservation of catfish fillets and to augment their quality.

## 2. Materials and Methods

### 2.1. Bee Chitosan Extraction

**Figure 1:**
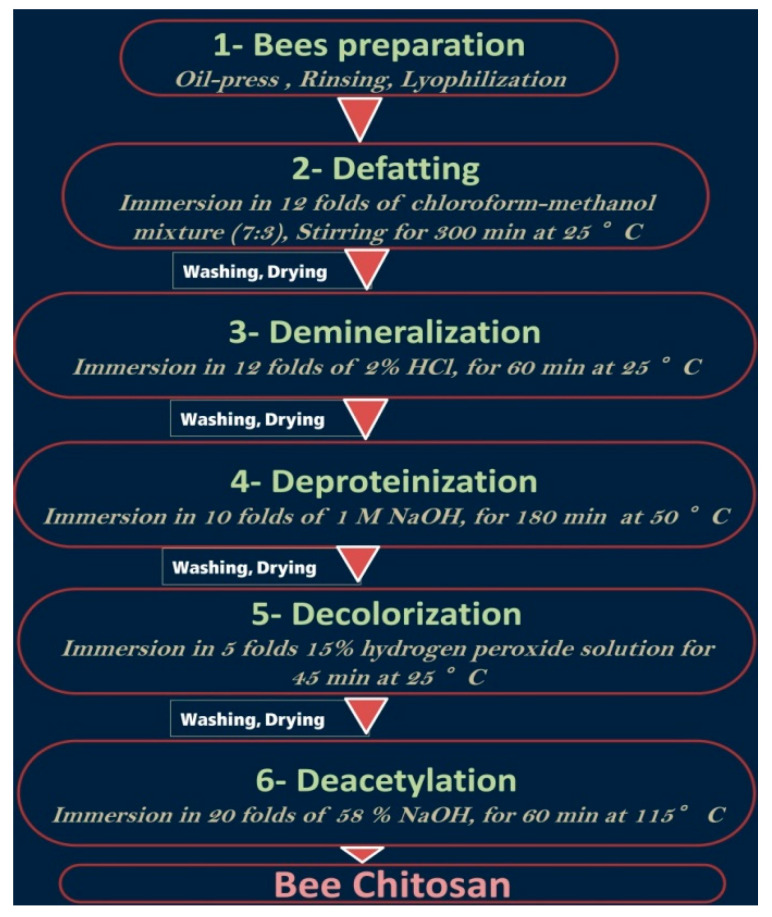
The applied protocol for bees’ chitosan extraction.

Corpses of honeybees (*Apis mellifera*) who died of natural causes were collected from 4 apiaries located in the Kafrelsheikh Governorate, Egypt, during March–October 2021. The bees’ corpses were combined after each collection, cleansed and frozen until usage to serve as the raw materials for chitosan extraction. After defrosting, the bee materials were subjected to oil pressing to eliminate most of their oils and proteins, then the skeletal residues were periodically washed using deionized water (DW) and freeze-dried [7]. The used protocol and steps for bee chitosan extraction are illustrated in Figure 1; the DW washing and lyophilization were repeated after each step.

For achieving bee chitosan extraction, nanoparticles (BCht) and solutions of Na-tripolyphosphate (NTPP; Sigma-Aldrich, MO, USA) with a 0.5% concentration (*w*/*v*) in DW and chitosan with a 0.1% concentration (*w*/*v*) in diluted acetic acid (1% concentration) were prepared and filtered, and their pH was adjusted to 5.3. In total, 20 mL of the NTPP solution was slowly dropped (via syringe needle at 350 µL/min rate) into 100 mL of the chitosan solution and was vigorously stirred throughout synthesis (725× *g*); the stirring continued after NTPP dropping for a further 100 min. The formed BCht was harvested via centrifugation (10,300× *g* for 32 min), washed with DW, re-centrifuged and lyophilized [34].

### 2.2. Propolis Extraction and SeNPs Synthesis

Crude propolis was attained from the above apiaries and was subjected to extraction with 10 folds (*v*/*w*) of 72% ethanol at room temperature (RT, 25 ± 2 °C) for 42 h, with occasional shaking (225× *g*). After filtration with Whatman No 2 paper, the extracted propolis (Pro) solution was statically preserved at 4 ± 1 °C for 24 h to precipitate the wax contents and re-filtered [35]. The filtered Pro solution was centrifuged and dried via vacuum evaporation (Buchi, Switzerland) at 40 °C until it dried. The dried Pro was re-constituted in an aqueous Tween 80 solution (2%) to obtain a concentration of 10% (*w*/*v*) [36].

For the biosynthesis of SeNPs with Pro, the freshly made extract with 1% concentration was dropped for mixing with a reaction solution that contained final concentrations from sodium selenite and ascorbic acid of 10 mM and 25 mM, respectively [12]. The mixture was stirred (220× *g*) at RT for 8 h, and the color change was observed from bale yellow to deep brownish orange. The Pro-mediated SeNPs were harvested via 10,500× *g* centrifugation for 35 min, washed with DW and ethanol then lyophilized. For analyzing plain SeNPs, the composite Pro/SeNPs were further washed with DW and ethanol (4 times each), centrifuged after each wash and lyophilized.

### 2.3. Products’ Physiognomies Characterization

#### 2.3.1. SPR (Surface Plasmon Resonance) Evaluation

The spectrum of Pro-mediated SeNPs was documented using UV-Visible spectrophotometry (UV-2450, Shimadzu, Japan); the absorbance measurement was conducted at a wavelength range of 220–550 nm.

#### 2.3.2. FTIR Analysis

The infrared analysis of produced molecules could validate their biochemical structures and interactions by detecting their biochemical bonding. Fourier transform infrared spectroscopy (FTIR; FTS 45, Biorad, Germany) was employed for assessing the infrared spectra of NCt, RA and NCt/RA/SeNPs. The transmittance of samples was appraised with a wavenumber in the range 400–4000 cm^−1^ after amalgamating each sample with 1% KBr [24]. The analysis was performed at 22 °C and at a resolution of 4 cm^−1^, each dried sample was finely ground and mixed well with anhydrous KBr, then amalgamated samples were positioned for FTIR analysis.

#### 2.3.3. Fourier-Transform Infrared Spectroscopy (FTIR) Analysis

The FTIR spectral analysis of extracted Pro, BCht, Pro/SeNPs and BCht/Pro/SeNPs nanocomposite were spectrophotometrically screened (JASCO 4100, Tokyo, Japan) with a wavenumber in the range 450–4000 cm^−1^. Powdered materials were homogeneously amalgamated with KBr and the transmittance spectrum was plotted for each powder.

#### 2.3.4. Electron Microscopy Analysis

TEM (JEOL, JEM-2100, Tokyo, Japan) and SEM (JSM IT100, JEOL, Tokyo, Japan) were employed for appraising the morphology, size, and structure of Pro-mediated SeNPs and BCht, respectively. The TEM conditions included the operation at an accelerating voltage of 180 kV, where the sonicated NP solution was mounted onto a grid of carbon-coated copper and then vacuum dried. The SEM imaging involved the operation of 20 kV accelerating voltage.

#### 2.3.5. Determination of Nanoparticles’ Zeta Potential (ζ) and Particle Size (Ps) Distribution

The nanoparticles’ size and charges of fabricated BNCt, Pro-synthesized SeNPs and their nanocomposites (BNCt/Pro/SeNPs) were assessed via the DLS protocol (Dynamic Light Scattering, Malvern Zetasizer, Malvern Instruments, Malvern, UK). The NP’s surface charges (ζ potential) and NP’s size distributions were analyzed in the dispersed NPs in the DW solution after their sonication.

### 2.4. Antibacterial Potentiality Assessments

The antibacterial potentialities of BCht, Pro, Pro/SeNPs, and the nanocomposites of these agents, were appraised qualitatively/quantitatively against standard bacterial strains, e.g., *Salmonella typhimurium-* ATCC 14028, *Escherichia coli* ATCC 25922, and *Staphylococcus aureus-* ATCC 25923. The bacterial microorganisms were propagated, subcultured and challenged at 37 ± 1 °C using nutrient broth (NB) and agar (NA) media (Difco Labs, Detroit, MI, USA).

#### 2.4.1. Qualitative Disc Diffusion Assay

The qualitative antimicrobial assay involved the measurement of appearing zones of growth inhibition (ZOI) following the “disc diffusion” method, which was performed by plating bacterial cultures onto NA media and putting impregnated paper discs (6.0 mm diameter) with 28 µL of every agent’s solution (with 10% concentration, *w*/*v*) on the inoculated plates’ surface. After the plates were incubated for 18–22 h, the ZOIs that appeared around the assay discs were measured. Ampicillin (Merck, Darmstadt, Germany) was used as the standard antibiotic to compare antibacterial activity, with similar challenge conditions.

#### 2.4.2. Quantitative Minimum Inhibitory Concentration (MIC) Assay

The described micro-dilution technique was operated to assess the MICs of nanocomposites and plain extracts [37] using the TPTC indicator (Triphenyl tetrazolium chloride, Merck, Germany) for confirming microbial survival. Sequential concentrations of 10–100 µg/mL, from every compound/composite in NB, were made using 96-well microplates. The wells were inoculated with ~2 × 10^7^ CFU/mL from each screened bacterial cell, and 0.5% (*w*/*v*) of TPTC was added. To verify the bactericidal action, samples from colorless wells were successively plated onto NA plates and incubated. The MICs were stated as the least concentration of individual agents that could prohibit the survival of specific bacteria in microplates and on NA plates.

#### 2.4.3. SEM Imaging of Antibacterial Action

The morphological deviations in *S. aureus* cells’ structure, after exposure to the BNCt/Pro/SeNPs nanocomposite solution (100 mg/L concentration), were screened through SEM imaging after exposure for 5 and 10 h and incubation aerobically at 37 °C, as described earlier [2]. The captured SEM micrographs were performed at ×10,000 magnification and an acceleration of 20 kV; capture depended on the emerged distortions in the cell’s morphology/structure.

### 2.5. Catfish Fillets’ Coating with Fabricated Compounds Edible Coatings

#### 2.5.1. Catfish Fillet Preparation

Thirty-eight alive African sharptooth catfish, *Clarias gariepinus*, Burchell, with a weight of ~460 ± 20 g per fish, were obtained from The Aquaculture Research Farm, Kafrelsheikh University, Egypt, and transported immediately at RT in water paths to the Seafood Processing Research Plant, Kafrelsheikh University. Fish were slaughtered, beheaded, skinned, filleted and cut to obtain fillet pieces of ~100 ± 4 g per piece (~6 × 10 × 1.5 cm^3^ dimensions). The study plan and performance were authenticated by the Committee of Aquatic Animal Care and Use in Research, Faculty of Aquatic and Fisheries Sciences, Kafrelsheikh University, Egypt with the approval No. IAACUC-KSU-30-2019.

#### 2.5.2. Preparation of Edible Coating (EC)

For the preparation of EC solutions, the screened compounds/composites (BCht, Pro, BCht/Pro, Pro/SeNPs and BCht/Pro/SeNPs) were dispersed at RT in autoclaved DW at 1.0% (*w*/*v*) concentration and sonicated until complete dissolution. Glycerol was then added as a plasticizer at 0.25% (*v*/*v*) into the solutions and vortexed for homogenous mixing [31]. The attained ECs were aseptically kept at RT until subsequent usage.

#### 2.5.3. Catfish Fillets Treatment

Six groups were analyzed throughout the coating experiments, including the control (DW coated), samples coated with BCht, Pro, Pro/SeNPs, Pro/BCht, and BCht/Pro/SeNPs. The fish fillet groups consisted of 25 pieces per group, with matching pieces’ sizes. The control group was dipped in sterilized DW without any additives, whereas each other group was immersed in one EC solution for 2 min, drained for 5 min, re-immersed in the EC for 1 min, and re-drained on metal nets at 8 ± 2 °C for 3 h. All experiments were conducted in an aseptic environment; all samples were packed in polyethylene trays and stored at 4 ± 1 °C for consequential quality assessments [32]. The chemical, microbiological and sensorial assessments were carried out at zero-day and after 7 days of cold storage.

### 2.6. Analysis of Coated Fillets’ Parameters

#### 2.6.1. Microbiological Examination

The control and EC-treated fillets were aseptically sampled (20 g/sample) and each sample was submerged into 180 mL of 0.1% buffered peptone medium (LAB-M, Lancashire, UK), which was then put in a Stomacher Bag and homogenized (3 min, Seward Stomacher 400, Norfolk, UK). Successive dilutions from fillets’ homogenates were prepared in NB and assessed for microbial counting after plating onto solidified media as described in the standardized protocols below:

ISO 4833-1:2013: “Enumeration of total aerobic microorganisms of colony count at 30 °C” [38].
ISO 17410:2019: “Enumeration of total microbial psychrotrophic organisms” [39].
ISO 21527-1:2008: “Enumeration of yeasts and molds” [40].

#### 2.6.2. Chemical Examinations

The measurements of spoilage chemical parameters in treated catfish fillets included the assessment of total volatile basic nitrogen (TVB-N) and thiobarbituric acid reactive substances (TBARS).

The TVB-N value measurement applied the method of hydro-distillation, as demonstrated within the method standard [41]. The used technique involved TVB extraction in alkaline suspension and titration of developed ammonia. Catfish samples (10 g) were homogenized with 20 mL of trichloroacetic acid (TCA, 7.5%, *w*/*v*) and filtered through Whatman paper. Steam hydro-distillation (UDK-6, VELP, Milan, Italy) was performed on filtrates after the addition of an alkaline NaOH solution (3 mL of 10%, *w*/*v*), then the distillates were gathered into a boric acid solution (4%, *w*/*v*) and amended with 1.1 mL of mixed indicator (1 methylene blue: 2 methyl red). The titration of the attained solution was conducted using H_2_SO_4_ (0.025 N).

TBARS determination was conducted via a spectrophotometer (Shimadzu, Kyoto, Japan) with an optical density wavelength (absorption strength) of 532 nm [42]. Fillet samples (10 g) were homogenized (IKA Homogenizer, Wilmington, NC, USA) with TCA (11%) for 70 s at 11,100 rpm, cooled in an ice bath for 60 sec, and homogenized again for a further 70 s. After the homogenates’ filtration through filter paper, an equal volume of thiobarbituric acid solution (20 mM) was added to the filtrates and incubated at RT in dark conditions for 20 h. The resulting solution absorbance at 532 nm was measured and calculated as mg of malondialdehyde (MDA)/kg.

#### 2.6.3. Sensory Analysis

The sensorial analysis of coated catfish fillets after 7 days of cold storage involved the assessments of samples’ appearance, odor, color and overall quality [32]. The evaluation board consisted of 9 females and 4 males with educational experiences in seafood judgment. The panelists assessed the EC fillets on a 9-point scale ranging from 9 (exceedingly good) to 1 (exceedingly poor). Three assessments were performed by each panelist after changing the samples’ positions and codes. The means of panelists’ scores were calculated to provide the final judgments.

### 2.7. Statistical Analysis

Triplicates of all experiments were performed (three assessments for each analysis); their means ± S.D. (standard deviations) were calculated with Microsoft Excel 365. The *t*-test and ANOVA of SPSS software (SPSS V-11.5, Chicago, IL, USA) were applied to statistically analyze the data significances at *p* ≤ 0.05. Using a mixed linear model with SPSS for the microbiological and chemical analysis of coated fillets, the treatments were set as the fixed factors for the model, whereas in the sensorial analysis, the treatments and storage time (zero and 7 days) were considered fixed factors in this model.

## 3. Results

### 3.1. Bee Chitosan

Bee chitosan was effectively gathered from insects’ bodies; the final polymer powders had a yellowish-white color, a molecular weight of 57.9 kDa, a deacetylation degree of 87.6%, and a solubility of 98.4% in 1% acetic acid solution.

### 3.2. Selenium Nanoparticles Biosynthesis Using Propolis Extract

**Figure 2:**
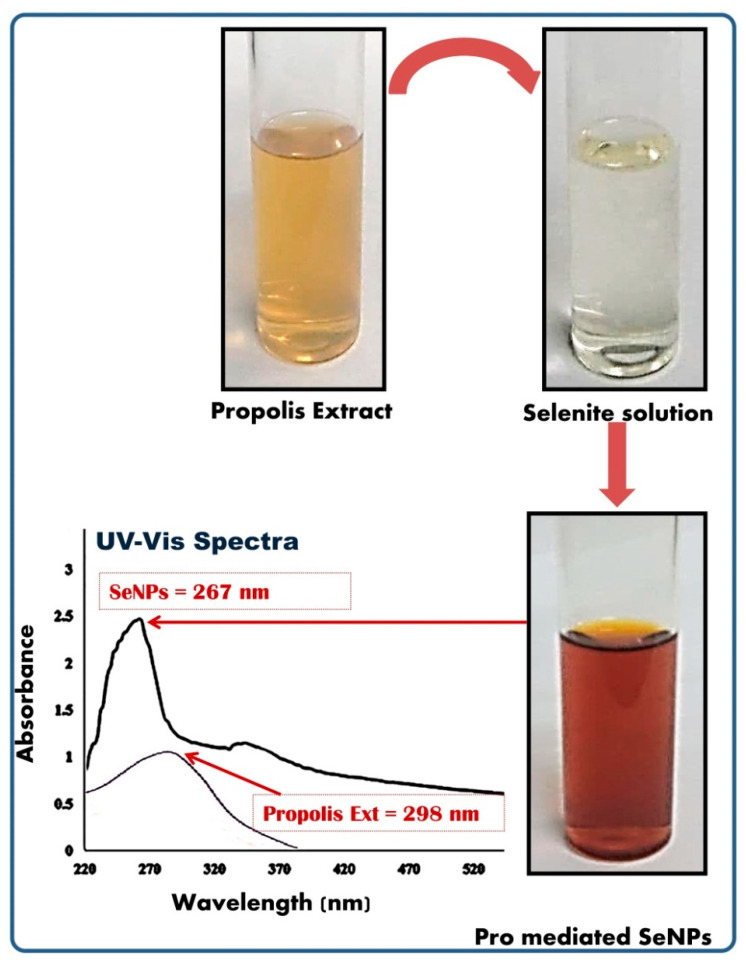
Visual appearance and UV-Vis spectrum of biosynthesized SeNPs using propolis extract.

The Pro extract could promisingly reduce Se ions to generate SeNPs, as shown by the alteration of the Se solution’s color from clear to brownish, which was easily noticeable from direct visual observation (Figure 2). The maximum UV-Vis absorbance (λ_max_) for the SeNP solution was recorded at 267 nm, whereas the λ_max_ for plain Pro was detected at 298 nm (Figure 2).

### 3.3. Structural Analysis of Synthesized Molecules

#### 3.3.1. FTIR Analysis

**Figure 3:**
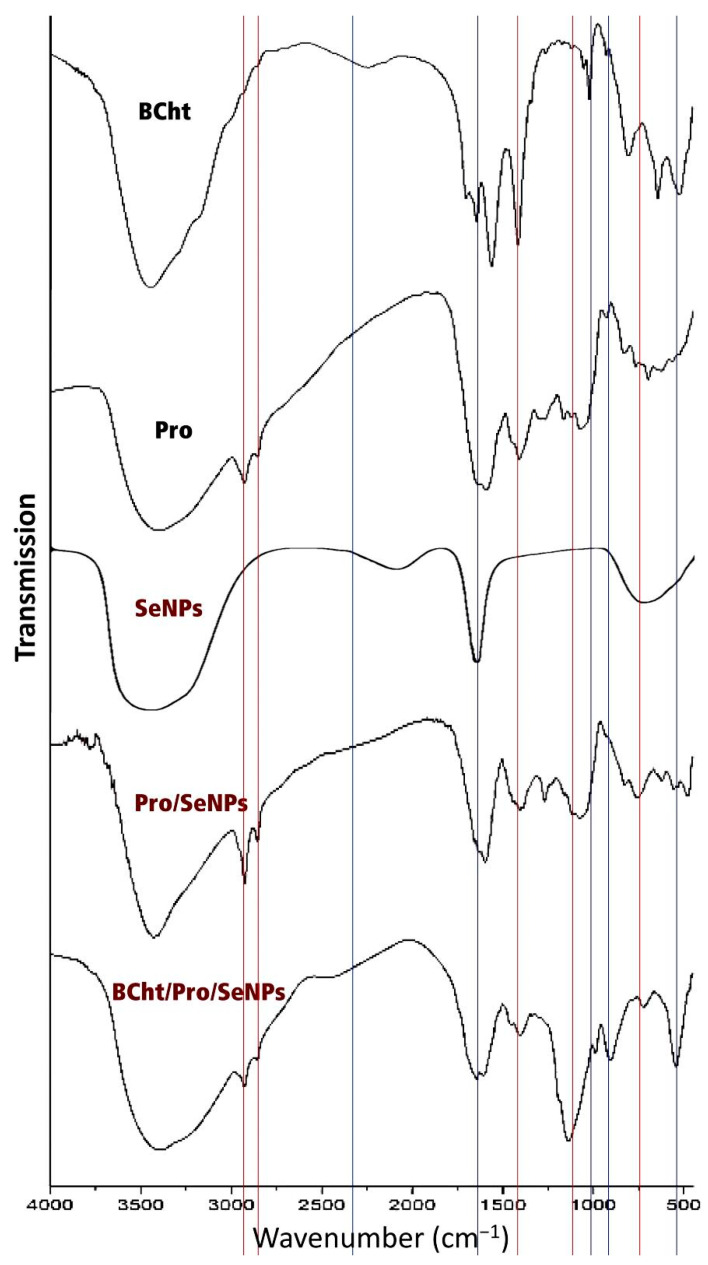
FTIR spectra of bee chitosan nanoparticles (BCht), propolis (Pro), Pro with synthesized SeNPs (Pro/SeNPs) and BCht/Pro/SeNPs nanocomposite.

The infrared analysis of synthesized molecules (e.g., BCht, Pro, Pro/SeNPs and BCht/Pro/SeNPs) was conducted to evaluate the main biochemical groups/ponds in these molecules that influence their activity and potentiality for generating a nanocomposite (Figure 3). The key designative peaks in the BCht spectrum (Figure 3-BCht) were detected at 1109 cm^−1^ (C-O), 1404 cm^−1^ (amide III, C-N), 1637 cm^−1^ (amide I, C=O), 2313 cm^−1^ (C-H), and 3455 cm^−1^ (NH_2_), respectively. Furthermore, the C-O bending and C-H stretching vibrations could be observed at 1066 cm^−1^, respectively. The physical interaction among the –NH group in the chitosan and TPP could be detected and appeared to peak at 3241 cm^−1^.

The FTIR spectrum of Pro showed the key designative groups/bonds in the extract (Figure 3-Pro). The peaks’ values around 2937 and 2846 cm^−1^ are assumingly attributed to the presence of a lignin polymer. The peak around 1462 cm^−1^ is indicative of the carboxyl group, whereas the peaks at 1028 and 732 cm^−1^ could represent the phenolic groups.

The spectrum of plain SeNPs (Figure 3-SeNPs) showed minimal functional groups due to the removal of most biological matter attached to the NPs during washing, whereas the FTIR analysis of the Pro/SeNPs spectrum (Figure 3-Pro/SeNPs) provided the potential molecules responsible for SeNPs synthesis/stabilizing. Results indicated that the Pro-containing compounds, which are rich with O-H, N-H, C-C and COOH, were the key agents for SeNP interaction. From them, the aliphatic saturated C-H stretching was detected at 2935 cm^−1^, and the peak at 1462 cm^−1^ indicated the CH_2_/CH_3_ of the carboxylate group and/or aliphatic compound. The peak at 1254 cm^−1^ corresponded to C-N and C-O-S in aromatic amine, whereas the peak at 1166 cm^−1^ indicated the SO_2_ and NO_2_ in sulphones. Additionally, the observed peaks between 1086 and 1041 cm^−1^ correspond to stretched C-H and C-O-C, arising from carbohydrate groups, while the peaks at 732 cm^−1^ and 557 cm^−1^ indicate the CH_2_ of hydrocarbons and the C-C=O of carboxylic acid.

The composites of BCht with Pro/SeNPs (Figure 3-BCht/Pro/SeNPs) had numerous biochemical bonds/groups from both composing agents (indicated by blue lines for groups derived from BCht and red lines for groups derived from Pro/SeNPs).

#### 3.3.2. Ultrastructure Analysis of Synthesized Nanoparticles

**Figure 4:**
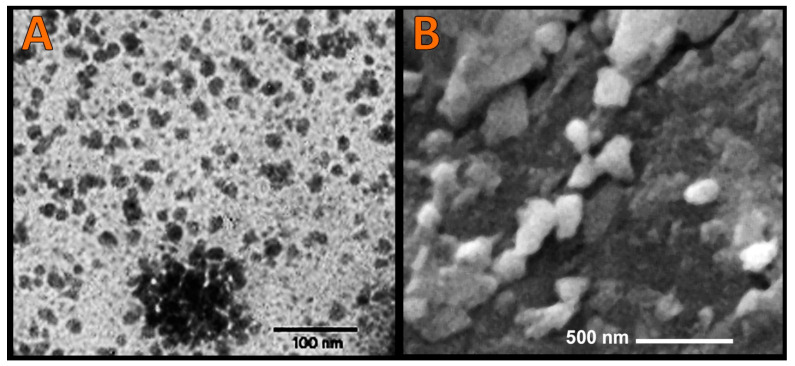
Ultrastructure of synthesized nanoparticles, including (**A**): transmission image of propolis-synthesized SeNPs and (**B**): scanning image of bee chitosan nanoparticles.

The physiognomies of synthesized nanoparticles/nanocomposites (BCht, Pro synthesized SeNPs and BNCt/Pro/SeNPs) were appraised via electron microscope imaging (Figure 4) and DLS analysis (Table 1). The NPs’ ultrastructure imaging via TEM indicated that Pro-synthesized SeNPs had a homogenous size range and good distribution, with spherical and semi-spherical shapes (Figure 4A). The BCht imaging via SEM proved the formation of polymer nanoparticles, which were heterogeneous in shape and had an estimated mean diameter of 159 nm (Figure 4B).

The DLS analysis validated the former results; the recorded particles’ mean diameters for BCht, Pro/SeNPs and BNCt/Pro/SeNPs were 151.9, 11.2 and 169.3 nm, respectively (Table 1). The BCht carried strong positive charges (+37.6 mV), which were slightly decreased to +32.2 mV after conjugation with Pro/SeNPs, whereas the SeNPs were negatively charged with −23.4 mV (Table 1).

### 3.4. Antibacterial Action of Nanocomposites

#### 3.4.1. In Vitro Antibacterial Potentialities

The antimicrobial activities that resulted from natural compound applications were proved, quantitatively and qualitatively, by the screened bacterial pathogens (Table 2). The entire compounds/composites, e.g., BCht, Pro, Pro/SeNPs and BCht/Pro/SeNPs, exhibited remarkable antibacterial in the following order: BCht < Pro < Pro/SeNPs < BCht/Pro/SeNPs. The bacterial strains were entirely susceptible to the examined agents. The bacterial sensitivities to the produced antimicrobial agents can be arranged in this order: Gram-positive < Gram-negative (*S. aureus* < *S. typhimurium* < *E. coli*). The antibacterial efficacy of the BCht/Pro/SeNPs composite was significantly stronger than standard antibiotic (ampicillin) regarding their ZOIs and MICs (Table 2).

#### 3.4.2. SEM Analysis of Challenged Bacteria with Nanocomposite

**Figure 5:**
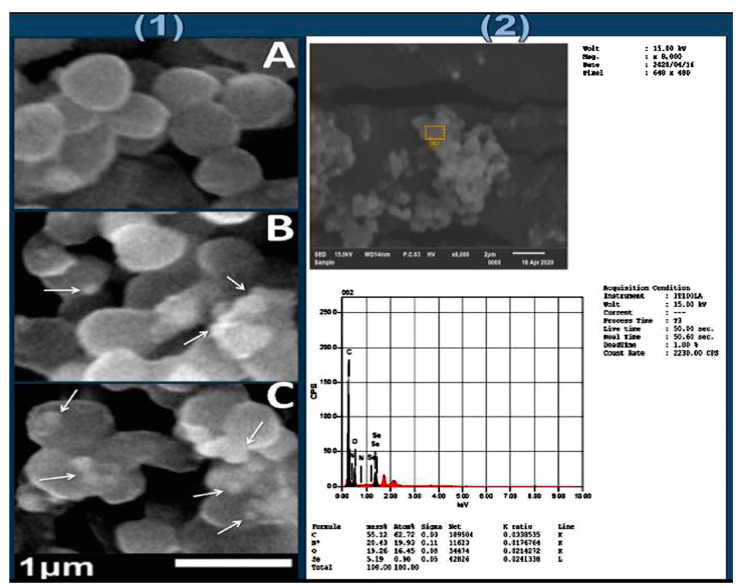
SEM (**1**) and EDS (**2**) of treated *Staphylococcus aureus* with BCht/Pro/SeNPs for 5 h (**1-B**) and 10 h (**1-C**) compared with control cells (**1-A**).

The SEM imaging of a bacterial pathogen (*S. aureus*) treated with a BCht/Pro/SeNP nanocomposite demonstrates its biocidal action for the destruction of bacterial cells (Figure 5). The control *S. aureus* cells, at the beginning of treatment, appeared with regular shapes and sizes of their structure/membranes; the cells had uniform, compact and smooth surfaces (Figure 5(1-A)). After 5 h from exposure to BCht/Pro/SeNPs, the *S. aureus* cells began to swell and partial lyse manifestation appeared in their structures (Figure 5(1-B)); many detectable NPs could be observed in the attachment/interaction with bacterial cells.

With the prolonged exposure of *S. aureus* cells to BCht/Pro/SeNP nanocomposites for 10 h, the cells were punitively damaged/destroyed; the attachments of NPs onto the cell surfaces became more detectable, as indicated by the arrows in Figure 5. The EDS analysis of treated *S. aureus* cells with BCht/Pro/SeNP nanocomposites indicated their elemental composition (Figure 5(2)), which involved the detection of Se ions in combination with C, N and O from the cell walls and from BCht and Pro constituents.

### 3.5. Biopreservation of Catfish Fillets with Natural Compounds

The consequences of catfish coatings with the produced antimicrobial compounds/composites (BCht, Pro, Pro/SeNPs, Pro/BCht, and BCht/Pro/SeNPs) on the microbial and chemical parameters of fish samples are demonstrated in Table 3. Compared to the zero-time control group, the control (water-dipped) samples exhibited extremely elevated values from the microbial group counts (e.g., total bacterial count, psychrophilic bacteria and yeast and molds) and from the chemical spoilage parameters (i.e., TVB-N and TBARS). The fillet samples coated with antimicrobial agents could significantly maintain their chemical and microbial qualities compared to the control (Table 3). The nanocomposites (Pro/SeNPs, Pro/BCht, and BCht/Pro/SeNPs) generally exhibited significantly higher efficacy for conserving a fillet’s qualities than plain molecules (BCht and Pro). Compared to the control, BCht/Pro/SeNPs was the most significantly effective formulation for decreasing the microbial counts and chemical parameter increments after 7 days of storage.

The BCht/Pro/SeNP coating reduced the counts of psychrophilic bacteria and yeast and molds to non-detectable levels after storage. The subsequent nanocomposite for reducing the microbial group count was the Pro/SeNPs, whereas the second-most effective nanocomposite for delaying the development of spoilage chemical parameters indicators was the Pro/BCht composite.

### 3.6. Sensorial Quality of Coated Catfish Fillets with Natural Compound

The sensorial consequences of catfish treatments with produced compound/composite-based edible coatings after cold storage (4 ± 1 °C) for 7 days are itemized in Table 4. Designating scores of ≥4.0 as the limit for sample acceptance, it is evident that the control (water-dipped) samples were rejected by panelists by evaluating their sensorial attributes after storage. On the contrary, all the coated fish fillets with produced compounds/composites (BCht, Pro, Pro/SeNPs, Pro/BCht, and BCht/Pro/SeNPs) were accepted for consumption after cold storage. The most promising and effective coating was that which contained 1% of the BCht/Pro/SeNPs nanocomposite, as it preserved the fillet’s sensorial qualities closest to the fresh product. Following BCht/Pro/SeNPs treatment, the second-most effective agent for keeping the qualities of the fillet’s appearance and odor was the Pro/SeNPs composite, whereas the third-best nanocomposite for maintaining the color and overall quality was the Pro/BCht (Table 4).

**Figure 6:**
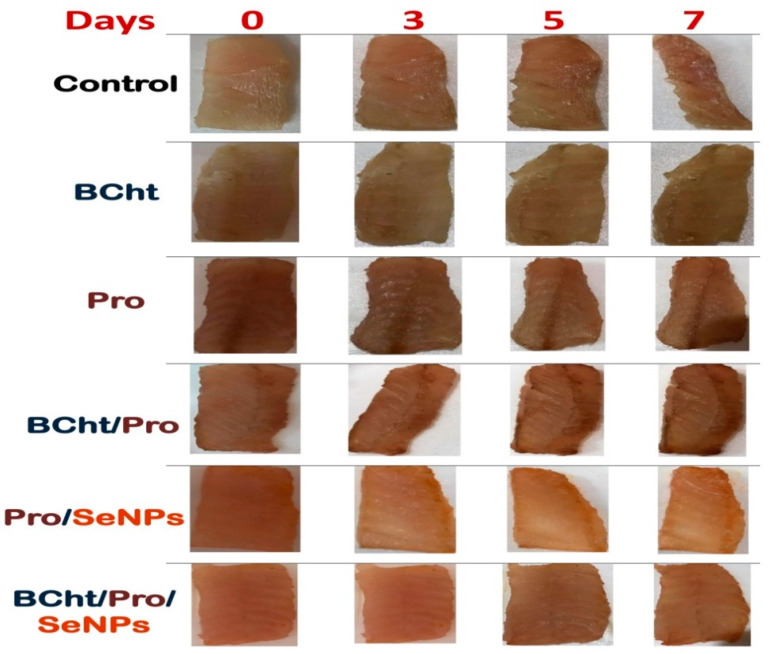
Manifestations of coated catfish fillets with bee chitosan nanoparticles (BCht), propolis (Pro), Pro with BCht, Pro with synthesized SeNPs (Pro/SeNPs) and BCht/Pro/SeNPs nanocomposite, compared to water-dipped (Control) samples, during cold storage for 7 days at 4 °C.

The manifestations of coated catfish fillets with the examined compounds/composites during their cold storage for 7 days at 4 °C are displayed in Figure 6. While the appearance and texture of control samples became unacceptable after 7 days, the coated samples, especially with BCht/Pro/SeNPs and Pro/SeNPs, could preserve their appearance, color and textures for the duration of the storage period (Figure 6).

## 4. Discussion

The achieved characteristics of bee-extracted chitosan were the desirable attributes for common chitosan, i.e., a deacetylation percentage of ≥70% and a low relative molecular weight [1]. The yield and characteristics of bee chitosan are comparable with insect chitosan, which has been produced in many other studies [8,10,11].

The assessment of NPs’ characteristics (e.g., size, structure and shape) is necessary for evaluating the particles’ properties; many methods have been applied, but UV-visible spectrophotometric assessment, FTIR analysis, X-ray diffraction, DLS and electron microscopy imaging are the most widely employed techniques [43]. As demonstrated by its characterization, the UV-vis assessment and visual observation of Pro-synthesized SeNPs could verify the high capability of Pro to reduce Se ions and transform them into SeNPs. The alteration of SeNPs’ solution color after Pro reduction is accredited to the SPR, which frequently recorded its λmax at ~265–275 nm [20,44,45,46].

The BCht spectrum (Figure 2-BCht) exhibited typical bonds/groups of standard chitosan (particularly from insects’ origin), which are documented in the literature [5,7,11], which strongly validated the successful extraction of chitosan from bee skeletons. The interactions between chitosan groups (e.g., –NH group) and TPP indicated successful BCht synthesis [47]. The Pro spectrum validated the occurrence of numerous functional groups (e.g., alcohols and polyphenols), which could interfere with the reduction, capping and stabilization of SeNPs [12,35,48]. The FTIR for the Pro/SeNPs was principally investigated to designate the main biological compounds in Pro that interfered in the synthesis/stabilizing of SeNPs. The shifted, emerged and disappeared bands of the Pro groups indicated their physiochemical interactions with SeNPs [12,45]. Additionally, the reducing powers and antioxidant activity of Pro molecules could enforce the reduction of Se to SeNPs, which was recently stated for AgNPs synthesis with Pro [49]. The BCht particles’ size was slightly increased after encapsulating Pro/SeNPs with a minor reduction in BCht surface positivity, which verified the efficiency of these nanopolymer particles for capping/encapsulating the conjugated molecules [34,45,49,50]. The slightly decreased positivity of BCht after conjugation with Pro/SeNPs may additionally appoint their physical capping of the nanocomposite rather than biochemical interactions with them [50]. The recorded zeta-potentialities (<−30 mV and >+30 mV) or inspected NPs and their DLS marks reflect their outstanding stability and dispersion in solutions [26]. These auspicious findings in the current research, particularly the raised stability and miniature sizes of BCht-based nanocomposites, agree with formerly demonstrated results that indicate the ability of varied chitosan forms to carry further bioactive molecules and biosynthesized nanoparticles [34,49].

Regarding the antibacterial actions of fabricated nanoparticles/nanocomposites, the Gram- species were more sensitive to their microbicidal action than the Gram^+^ species (*S. aureus*), which compliments the former studies which investigated biosynthesized SeNPs and their composites with chitosan [21,33,34]. For elucidating these findings, the elevated resistances of Gram^+^ bacteria to bactericidal nanomaterials were assumed to arise from the presence of a thick peptidoglycan protective layer in Gram^+^ membranes that comprises teichoic/lipoteichoic acids. The Gram- bacterial surfaces encompass definite proteins (porins) which enable the penetration of biocidal molecules (including nanopolymers and nanometals) into cells. The generated reactive oxygen species (ROS) from SeNPs can also diffuse more easily into Gram- interior cells and inactivate/destroy their vital components [45,51,52].

The SEM imaging and the DLS analysis could validate the strong attachment and interaction of BCht/Pro/SeNPs with *S. aureus* cells, which led to remarkable destruction and inactivation of cell viability. The *S. aureus* was chosen for SEM imaging as it exhibited the least susceptibility to NPs among the screened species. The observed instructive action of NPs here could be attributed to the synergistic acts of BCht, Pro and SeNPs. This was proved via DLS analysis, which agreed with former studies that investigated the actions of chitosan and its nanoparticles in disrupting the bacterial cell barriers and increasing their permeability [2,53]. The bioactivities of chitosan and its NPs were proven, especially their antimicrobial potentialities, which principally result from the intensified positive charges on their surfaces. The particles’ positivity enables their interaction with the surface of the microorganism’s cells and their interior fundamental components (RNA, DNA, proteins, enzymes, etc.), which leads to microbial inactivation and death [4].

The phyto-synthesized SeNP’s actions were additionally validated to destroy bacterial pathogen’s cells and inactivate their viability through their penetration of cells, inducement of irregular walls’ permeability and generation of ROS that lead to bacterial death [24,34,46]. The SeNPs’ antimicrobial action was enhanced by their conjugation with chitosan NPs [34,50], and the Pro-mediated SeNPs were confirmed to have more antimicrobial powers than the effects of its compositing ingredients [12]. This was also stated for further Pro-mediated nanometals, which possessed stronger microbicidal actions than their elemental ingredients [17,49]. Furthermore, the acquired synergism between these conjugated antimicrobial agents (BCht, Pro and SeNPs) has influential potentiality for inhibiting bacterial strains; the microbial cell cannot survive in the presence of multiple antimicrobial compounds with diverse actions and from diverse sources [34,52].

The estimated amounts of SeNPs that were applied during a fillet’s coating with BCht/Pro/SeNPs are ~250–300 μg/kg, which relies on the safe daily levels of Se consumption in humans (the demanded daily Se intake for the human body = 40–300 μg, whereas toxicity transpires at dosages higher than 3200 μg/day) [22,23]. The reported bioactivity, biotoxicity and bioavailability of Se particles are principally influenced by their structural forms; the SeNPs have abundantly lower toxicity with amplified bioavailability compared with bulk Se particles [19]. Additionally, the oral administrations of SeNPs in animal tests were recommended to augment the bioactivities of selenoenzymes, with a remarkable diminishing of their biotoxicity compared to diverse Se forms (e.g., sodium selenite, selenomethionine, or methylselenocysteine) [25]. These authenticated results can modify the dogma concerning Se elemental bioactivities and endorse SeNPs as safe, low-risk and effective sources to supplement Se with the lowest toxicity [19,25].

The recurrent SeNP applications in food packaging, preservation and processing could merit their diminished toxicity and elevated biosafety, which were formerly advocated to validate their high applicability and biosafety for direct contact and intake by humans [22,26]. Furthermore, as the incorporation of biomolecules (e.g., polysaccharides, phytocompounds, biopolymers…) through SeNP synthesis and stabilizing can afford more biosafety aspects for biosynthesized NPs [23], it can be expected that the conjugation of BCht with Pro-mediated SeNPs will possess preeminent compatibility and biosafety with minimal prospective toxicity. In addition, SeNPs, especially those synthesized with biogenic approaches, have lower toxicity than bulk selenium, and the SeNP’s antioxidant and radical scavenging potentialities were confirmed, which shows their suitability for chemopreventive applications (in cancer management) and as antioxidants (in food preservation approaches) [12,21,27]. The former could explain the elevated potentialities of SeNP-based ECs to preserve the chemical and microbial qualities of coated catfish fillets in this current study.

The TVB-N in fish results from their protein degradation, either by bacterial or autolytic enzyme actions; the acceptable limits for TVB-N in fish are preferably ≤35 mg/100 g [30,54]. However, the tendency of fish lipids to be oxidized is extremely high because of their contents of fatty acids (polyunsaturated) and the formation of TBARS [29]; the normal limits of TBARS for fish acceptability should be ≤3–4 mg malondialdehyde/kg [55].

The bacterial counts, and particularly the psychrophilic species, are extremely important for judging the quality of cold-storage fish because of the bacteria’s negative consequences on the sensorial and hygienic attributes of fish through the production of their diverse metabolic and toxicant compounds during growth, including biogenic amines, ketones, volatile sulfides and aldehydes [56]. The recommended limit of psychrophilic bacteria in stored fish should not exceed 4.0 log CFU/g [28], as this group possesses higher proteolytic and lipolytic bioactivities that directly affect the TBARS and TVB-N intensities in preserved fish.

The Pro radical scavenging and antioxidant activities were formerly proven with concentration-dependent traits [57,58], which are strongly involved in the protection of fish fillets from fat going rancid and protein decomposition. The bioactivities of Pro are principally attributed to its contents from phytochemicals (e.g., phenolic compounds; volatile aromatics, flavonoids and terpenes) [16,58]. The antimicrobial potentiality (toward diverse bacteria, yeast, parasites and molds) and antioxidant activity of many Pro polyphenols were demonstrated [12,16,57]. The Pro phytochemicals include the chrysin flavone, which has potent antimicrobial, antioxidant and anticancer powers [59,60]; the chrysin antimicrobial attributes are based on the capability for destroying the integrity of cell walls/membranes. Further, many other Pro polyphenols (e.g., ferulic acid, caffeic acid, and p-coumaric acid) were reported to disturb DNA biosynthesis in microbial and cancer cells [61]. Genistein is another isoflavone constituent in Pro, with extraordinary antioxidant and chemotherapeutic activities, which enable its applications for treating inflammation, cancers and microbial infections from mycotic and bacterial origins [48,62]. The flavonoid pinocembrin was also abundant in Pro, with numerous pharmacological activities including its anti-inflammatory antioxidant and wide antibacterial powers [63,64]. The Pro also encompasses glycolic acid, glycerol and vanillin, which are widely employed in plentiful cosmetic and food products, which is attributable to their capabilities as antimicrobial, anti-aging and antioxidant agents [16].

Pro and its derivatives can effectively kill bacterial cells through diverse mechanisms, e.g., via direct interaction with their cell walls and components or through modification of host cells’ immune responses [65]. The proposed mechanisms for Pro’s antimicrobial actions are the obstruction of cell division, proteins and nucleic acids synthesis, impediment of cytoplasmic membranes’ functions and permeability, bacteriolysis, reduction of bacterial resistance, decrement of biofilms formation ability, and inhibition of energy pathways [66]. Moreover, the Pro-mediated SeNPs were reported to possess extra antimicrobial and antioxidant potentialities than their compositing compounds [12].

The polymeric nature and diminished particles’ size of BCht enforced its antioxidant and antimicrobial actions in ECs, especially after conjugation with the further bioactive molecules Pro and SeNPs. The chitosan NPs was formerly applied for constructing ECs in conjugation with other plant extracts and nanometals, which were reported to strengthen their combined microbicidal, anti-oxidative and surface barring abilities [5,31,32]. The nanocomposite ECs based on chitosan NPs were recommended for protecting foodstuffs from causative factors spoilage, either externally (e.g., from free radicals and O_2_ attack, external contaminations, …) or internally (e.g., from lipid oxidation, microbiological loads, enzymatic actions, …), which give more rationales to these natural and biosafe EC components, especially after conjugation with Pro constituents [5,34,66].

## 5. Conclusions

The fabrication of BCht from bee waste and biosynthesized SeNPs with Pro was successfully achieved. The BCht, Pro-mediated SeNPs and BNCt/Pro/SeNPs had mean diameters of 151.9, 11.2 and 169.3 nm, respectively. All the produced agents/composites exhibited persuasive antibacterial powers toward foodborne pathogens; BNCt/Pro/SeNPs were the most forceful toward all bacterial strains. The constructed ECs from produced compounds/composites were effective for preserving catfish fillets during cold storage. The microbiological, chemical and sensorial attributes of EC-treated fillets indicated the nanocomposite efficiency for protecting fish from microbial growth and the progress of chemical spoilage indicators and maintaining the sensorial quality of treated stored fillets. The ECs based on BNCt, Pro/SeNPs and their nanocomposites can be endorsed for prospective employment in the biopreservation of various seafoods.

## Figures and Tables

**Table 1:** Nanoparticles’ size and charges of fabricated bee nanochitosan (BNCt), propolis-synthesized SeNPs (Pro-SeNPs) and their nanocomposites (BNCt/Pro/SeNPs).

Nanoparticles	Particle Size Range (nm)	Particle Size Mean (nm)	Zeta Potential (mV)
BNCt	43.19–242.56	151.85	+37.6
Pro-SeNPs	4.72–31.73	11.21	−23.4
BNCt/Pro/SeNPs	59.51–304.63	169.28	+32.2

**Table 2:** Antimicrobial activity of bee chitosan nanoparticles (BCht), propolis (Pro), Pro/SeNPs and BCht/Pro/SeNPs.

Antimicrobial Agents	Antimicrobial Assay *					
	*Escherichia coli*		*Staphylococcus aureus*		*Salmonella typhimurium*	
	ZOI **	MIC ***	ZOI	MIC	ZOI	MIC
BCht	18.3 ± 1.3 ^a^	50.0	16.4 ± 0.8 ^a^	57.5	17.6 ± 1.2 ^a^	52.5
Pro	23.1 ± 1.6 ^b^	35.0	19.1 ± 1.3 ^b^	45.0	22.4 ± 1.8 ^b^	35.0
Pro/SeNPs	26.6 ± 1.9 ^b^	32.5	24.2 ± 1.8 ^c^	37.5	27.1 ± 2.2 ^c^	30.0
BCht/Pro/SeNPs	33.4 ± 2.4 ^c^	27.5	28.9 ± 2.1 ^d^	30.0	32.9 ± 2.6 ^d^	25.0
Ampicillin	25.8 ± 1.7 ^b^	37.5	23.5 ± 3.7 ^c^	47.5	24.4 ± 2.1 ^b^	40.0

* Different superscript letters in one column indicate significant difference at *p* ≤ 0.05, ** ZOI: Mean diameter of inhibition zones in mm ± standard deviation, *** MIC: minimal inhibitory concentrations (mg/L).

**Table 3:** Microbial and chemical attributes of coated fillets with antimicrobial compounds, after cold storage for 7 days at 4 °C.

Coating Material *	Assessment Attributes **				
	Microbial Quality (log CFU/g)			Chemical Quality (mg/kg)	
	Total Count	Psychrophilic Bacterial	Yeast and Molds	TVB-N	TBARS
Control (zero day)	4.72 ± 0.65 ^a^	3.24 ± 0.62 ^a^	2.26 ± 0.63 ^a^	110.07 ± 2.17 ^a^	0.41 ± 0.04 ^a^
BCht	2.89 ± 0.37 ^b^	1.95 ± 0.48 ^b^	1.38 ± 0.44 ^b^	169.94 ± 2.81 ^b^	0.95 ± 0.13 ^b^
Pro	2.07 ± 0.43 ^bc^	1.62 ± 0.53 ^b^	1.16 ± 0.55 ^b^	173.26 ± 3.54 ^b^	1.21 ± 0.10 ^b^
Pro/SeNPs	1.51 ± 0.29 ^c^	1.08 ± 0.46 ^b^	ND ***	164.51 ± 1.68 ^b^	0.86 ± 0.09 ^bc^
Pro/BCht	1.86 ± 0.42 ^c^	1.29 ± 0.61 ^b^	ND	132.15 ± 3.21 ^c^	0.78 ± 0.08 ^c^
BCht/Pro/SeNPs	1.05 ± 0.19 ^c^	ND	ND	118.47 ± 2.43 ^d^	0.65 ± 0.07 ^c^
Control	8.68 ± 1.34 ^d^	6.06 ± 1.03 ^c^	4.94 ± 0.94 ^c^	258.51 ± 4.37 ^e^	2.18 ± 0.16 ^d^

* The coating materials contained 1% (*w*/*v*) from bee chitosan nanoparticles (BCht), propolis extract (Pro), Pro/SeNPs and BCht/Pro/SeNPs composite, whereas the control samples were dipped in sterilized water, ** Different superscript letters in one column indicate significant difference at *p* ≤ 0.05, *** ND: not detectable.

**Table 4:** Sensory evaluation of preserved fish fillets with coated fillets with antimicrobial compounds, after cold storage for 7 days at 4 °C (Scores/9).

Agents	Appearance	Odor	Color	Overall Quality
Control	3.6	2.4	3.9	3.3
BCht	6.3	6.9	6.4	6.5
Pro	6.6	7.9	7.1	7.3
Pro/SeNPs	7.5	8.2	8.1	7.8
Pro/BCht	7.1	7.4	8.3	7.9
BCht/Pro/SeNPs	8.7	8.5	8.3	8.8

## Data Availability

The data presented in this study are available on request from the corresponding author.

## References

[1] Rinaudo M. (2006). Chitin and chitosan: Properties and applications. Prog. Polym. Sci..

[2] Tayel A.A. (2016). Microbial chitosan as a biopreservative for fish sausages. Int. J. Biol. Macromol..

[3] Zhang M., Zhang F., Li C., An H., Wan T., Zhang P. (2022). Application of Chitosan and Its Derivative Polymers in Clinical Medicine and Agriculture. Polymers.

[4] Radwan-Pragłowska J., Piątkowski M., Deineka V., Janus Ł., Korniienko V., Husak E., Holubnycha V., Liubchak I., Zhurba V., Sierakowska A. (2019). Chitosan-based bioactive hemostatic agents with antibacterial properties—synthesis and characterization. Molecules.

[5] Perinelli D.R., Fagioli L., Campana R., Lam J.K., Baffone W., Palmieri G.F., Casettari L., Bonacucina G. (2018). Chitosan-based nanosystems and their exploited antimicrobial activity. Eur. J. Pharm. Sci..

[6] Papagiannopoulos A., Sotiropoulos K. (2022). Current Advances of Polysaccharide-Based Nanogels and Microgels in Food and Biomedical Sciences. Polymers.

[7] Marei N.H., El-Samie E.A., Salah T., Saad G.R., Elwahy A.H. (2016). Isolation and characterization of chitosan from different local insects in Egypt. Int. J. Biol. Macromol..

[8] Hahn T., Roth A., Ji R., Schmitt E., Zibek S. (2020). Chitosan production with larval exoskeletons derived from the insect protein production. J. Biotechnol..

[9] Rossi M., Marrazzo P. (2021). The potential of honeybee products for biomaterial applications. Biomimetics.

[10] Marei N., Elwahy A.H., Salah T.A., El Sherif Y., El-Samie E.A. (2019). Enhanced antibacterial activity of Egyptian local insects’ chitosan-based nanoparticles loaded with ciprofloxacin-HCl. Int. J. Biol. Macromol..

[11] Luo Q., Wang Y., Han Q., Ji L., Zhang H., Fei Z., Wang Y. (2019). Comparison of the physicochemical, rheological, and morphologic properties of chitosan from four insects. Carbohydr. Polym..

[12] Shubharani R., Mahesh M., Yogananda Murthy V. (2019). Biosynthesis and characterization, antioxidant and antimicrobial activities of selenium nanoparticles from ethanol extract of Bee Propolis. J. Nanomed. Nanotechnol..

[13] Šturm L., Ulrih N.P. (2020). Advances in the Propolis Chemical Composition between 2013 and 2018. A Review. eFood.

[14] Sforcin J.M., Bankova V. (2011). Propolis: Is there a potential for the development of new drugs?. J. Ethnopharmacol..

[15] Ristivojevic´ P., Trifkovic´ J., Andric´ F., Milojkovic´-Opsenica D. (2015). Poplar-type propolis: Chemical composition, botanical origin and biological activity. Nat. Prod. Commun..

[16] Bouchelaghem S. (2022). Propolis characterization and antimicrobial activities against *Staphylococcus aureus* and *Candida albicans*: A review. Saudi J. Biol. Sci..

[17] Botteon C.E.A., Silva L.B., Ccana-Ccapatinta G.V., Silva T.S., Ambrosio S.R., Veneziani R.C.S., Bastos J.K., Marcato P.D. (2021). Biosynthesis and characterization of gold nanoparticles using Brazilian red propolis and evaluation of its antimicrobial and anticancer activities. Sci. Rep..

[18] Singh J., Dutta T., Kim K.H., Rawat M., Samddar P., Kumar P. (2018). Green synthesis of metals and their oxide nanoparticles: Applications for environmental remediation. J. Nanobiotechnol..

[19] Husen A., Siddiqi K.S. (2014). Plants and microbes assisted selenium nanoparticles: Characterization and application. J. Nanobiotechnol..

[20] Srivastava N., Mukhopadhyay M. (2015). Green synthesis and structural characterization of selenium nanoparticles and assessment of their antimicrobial property. Bioprocess Biosyst. Eng..

[21] Menon S., Agarwal H., Rajeshkumar S., Rosy P.J., Shanmugam V.K. (2020). Investigating the antimicrobial activities of the biosynthesized selenium nanoparticles and its statistical analysis. Bionanoscience.

[22] Skalickova S., Milosavljevic V., Cihalova K., Horky P., Richtera L., Adam V. (2017). Selenium nanoparticles as a nutritional supplement. Nutr. J..

[23] Dash K.K., Deka P., Bangar S.P., Chaudhary V., Trif M., Rusu A. (2022). Applications of inorganic nanoparticles in food packaging: A Comprehensive Review. Polymers.

[24] Huang X., Chen X., Chen Q., Yu Q., Sun D., Liu J. (2016). Investigation of functional selenium nanoparticles as potent antimicrobial agents against superbugs. Acta Biomater..

[25] Zhao G., Wu X., Chen P., Zhang L., Yang C.S., Zhang J. (2018). Selenium nanoparticles are more efficient than sodium selenite in producing reactive oxygen species and hyper-accumulation of selenium nanoparticles in cancer cells generates potent therapeutic effects. Free Radic. Biol. Med..

[26] Bhattacharjee A., Bastu A., Bhattacharya S. (2019). Selenium nanoparticles are less toxic than inorganic and organic selenium in mice in vivo. Nucleus.

[27] Zhai X., Zhang C., Zhao G., Stoll S., Ren F., Leng X. (2017). Antioxidant capacities of the selenium nanoparticles stabilized by chitosan. J. Nanobiotechnol..

[28] Navarro-Segura L., Ros-Chumillas M., Martínez-Hernández G.B., López-Gómez A. (2020). A new advanced packaging system for extending the shelf life of refrigerated farmed fish fillets. J. Sci. Food Agric..

[29] Cheng J.H., Sun D.W., Zeng X.A., Liu D. (2015). Recent advances in methods and techniques for freshness quality determination and evaluation of fish and fish fillets: A review. Crit. Rev. Food Sci. Nutr..

[30] Bernardo Y.A., Rosario D.K., Delgado I.F., Conte-Junior C.A. (2020). Fish Quality Index Method: Principles, weaknesses, validation, and alternatives—A review. Compr. Rev. Food Sci. Food Saf..

[31] Tayel A.A., Elzahy A.F., Moussa S.H., Al-Saggaf M.S., Diab A.M. (2020). Biopreservation of shrimps using composed edible coatings from chitosan nanoparticles and cloves extract. J. Food Qual..

[32] Alsaggaf M.S., Moussa S.H., Tayel A.A. (2017). Application of fungal chitosan incorporated with pomegranate peel extract as edible coating for microbiological, chemical and sensorial quality enhancement of Nile tilapia fillets. Int. J. Biol. Macromol..

[33] Gad H.A., Tayel A.A., Al-Saggaf M.S., Moussa S.H., Diab A.M. (2021). Phyto-fabrication of selenium nanorods using extract of pomegranate rind wastes and their potentialities for inhibiting fish-borne pathogens. Green Process Synth..

[34] Alghuthaymi M., Diab A., Elzahy A., Mazrou K., Tayel A.A., Moussa S.H. (2021). Green biosynthesized selenium nanoparticles by cinnamon extract and their antimicrobial activity and application as edible coatings with nano-chitosan. J. Food Qual..

[35] Elnakady Y., Rushdi A., Franke R., Abutaha N., Ebaid H., Baabbad M., Omar M.O.M., Al Ghamdi A.A. (2017). Characteristics, chemical compositions and biological activities of propolis from Al-Bahah. Saudi Arabia. Sci. Rep..

[36] Tayel A.A., El-Tras W.F. (2012). Plant extracts as potent biopreservatives for *Salmonella typhimurium* control and quality enhancement in ground beef. J. Food Saf..

[37] Tayel A.A., Moussa S.H., Opwis K., Knittel D., Schollmeyer E., Nickisch- Hartfiel A. (2010). Inhibition of microbial pathogens by fungal chitosan. Int J Biol Macromol..

[38] (2013). Microbiology of the Food Chain—Horizontal Method for the Enumeration of Microorganisms—Part 1: Colony Count at 30 Degrees C by the Pour Plate Technique.

[39] (2019). Microbiology of the Food Chain—Horizontal Method for the Enumeration of Psychrotrophic Microorganisms.

[40] (2008). Microbiology of Food and Animal Feeding Stuffs—Horizontal Method for the Enumeration of Yeasts and Moulds—Part 1: Colony Count Technique in Products with Water Activity Greater than 0.95.

[41] Official Journal of the European Communities (1995). Commission Decision of 8 March 1995. Fixing the Total Volatile Basic Nitrogen (TVB-N) Limit Values for Certain Categories of Fishery Products and Specifying the Analysis Methods to be Used. No L 97/84. (95/149/EC). https://eur-lex.europa.eu/legal-content/EN/TXT/PDF/?uri=CELEX:31995D0149&from=fr..

[42] Ke P.J., Cervantes E., Robles-Martinez C. (1984). Determination of thiobarbituric acid reactive substances (TBARS) in fish tissue by an improved distillation spectrophotometric method. J Sci. Food Agric..

[43] Shang L., Nienhaus K., Nienhaus G.U. (2014). Engineered nanoparticles interacting with cells: Size matters. J. Nanobiotechnol..

[44] Menon S., Shrudhi Devi K.S., Agarwal H., Shanmugam V.K. (2019). Efficacy of biogenic selenium nanoparticles from an extract of ginger towards evaluation on anti-microbial and anti-oxidant activities. Colloids Interface Sci. Commun..

[45] Al-Saggaf M.S., Tayel A.A., Ghobashy M.O., Alotaibi M.A., Alghuthaymi M.A., Moussa S.H. (2020). Phytosynthesis of selenium nanoparticles using the costus extract for bactericidal application against foodborne pathogens. Green Process Synth..

[46] ElSaied B.E., Diab A.M., Tayel A.A., Alghuthaymi M.A., Moussa S.H. (2021). Potent antibacterial action of phycosynthesized selenium nanoparticles using *Spirulina platensis* extract. Green Process Synth..

[47] El Rabey H.A., Almutairi F.M., Alalawy A.I., Al-Duais M.A., Sakran M.I., Zidan N.S., Tayel A.A. (2019). Augmented control of drug-resistant *Candida* spp. via fluconazole loading into fungal chitosan nanoparticles. Int. J. Biol. Macromol..

[48] Gargouri W., Osés S.M., Fernández-Muiño M.A., Sancho M.T., Kechaou N. (2019). Evaluation of bioactive compounds and biological activities of Tunisian propolis. LWT..

[49] Al-saggaf M.S. (2021). Formulation of Insect Chitosan Stabilized Silver Nanoparticles with Propolis Extract as Potent Antimicrobial and Wound Healing Composites. Int. J. Polym. Sci..

[50] Salem M.F., Abd-Elraoof W.A., Tayel A.A., Alzuaibr F.M., Abonama O.M. (2022). Antifungal application of biosynthesized selenium nanoparticles with pomegranate peels and nanochitosan as edible coatings for citrus green mold protection. J. Nanobiotechnol..

[51] Sonkusre P., Cameotra S.S. (2017). Biogenic selenium nanoparticles induce ROS-mediated necroptosis in PC-3 cancer cells through TNF activation. J. Nanobiotechnol..

[52] Breijyeh Z., Jubeh B., Karaman R. (2020). Resistance of Gram-negative bacteria to current antibacterial agents and approaches to resolve it. Molecules.

[53] Bekhit A.E., Holman B.W., Giteru S.G., Hopkins D.L. (2021). Total volatile basic nitrogen (TVB-N) and its role in meat spoilage: A review. Trends Food Sci. Technol..

[54] Baranwal J., Barse B., Fais A., Delogu G.L., Kumar A. (2022). Biopolymer: A sustainable material for food and medical applications. Polymers.

[55] Inoue T., Ando K., Kikugawa K. (1998). Specific determination of malonaldehyde by N-methyl-2- phenylindole or thiobarbituric acid. J. Am. Oil Chem. Soc..

[56] Gounot A.M. (1986). Psychrophilic and psychrotrophic microorganisms. Experientia.

[57] Ahn M.R., Kumazawa S., Usui Y., Nakamura J., Matsuka M., Zhu F., Nakayama T. (2007). Antioxidant activity and constituents of propolis collected in various areas of China. Food Chem..

[58] Bankova V., Popova M., Trusheva B. (2014). Propolis volatile compounds: Chemical diversity and biological activity: A review. Chem. Cent. J..

[59] Celińska-Janowicz K., Zaręba I., Lazarek U., Teul J., Tomczyk M., Pałka J., Miltyk W. (2018). Constituents of propolis: Chrysin, caffeic acid, p-coumaric acid, and ferulic acid induce PRODH/POX-dependent apoptosis in human tongue squamous cell carcinoma cell (CAL-27). Front. Pharmacol..

[60] Mani R., Natesan V. (2018). Chrysin: Sources, beneficial pharmacological activities, and molecular mechanism of action. Phytochem. Lett..

[61] Neto M.R., Tintino S.R., da Silva A.R., do Socorro Costa M., Boligon A.A., Matias E.F., de Queiroz Balbino V., Menezes I.R., Coutinho H.D. (2017). Seasonal variation of Brazilian red propolis: Antibacterial activity, synergistic effect and phytochemical screening. Food Chem. Toxicol..

[62] Choi H., Park J.S., Kim K.M., Kim M., Ko K.W., Hyun C.G., Ahn J.W., Seo J.H., Kim S.Y. (2018). Enhancing the antimicrobial effect of genistein by biotransformation in microbial system. Ind. Eng. Chem. Res..

[63] Rasul A., Millimouno F.M., Ali Eltayb W., Ali M., Li J., Li X. (2013). Pinocembrin: A novel natural compound with versatile pharmacological and biological activities. Biomed. Res. Int..

[64] Tundis R., Frattaruolo L., Carullo G., Armentano B., Badolato M., Loizzo M.R., Aiello F., Cappello A.R. (2019). An ancient remedial repurposing: Synthesis of new pinocembrin fatty acid acyl derivatives as potential antimicrobial/anti-inflammatory agents. Nat. Prod. Res..

[65] Almuhayawi M.S. (2020). Propolis as a novel antibacterial agent. Saudi Sci. J. Biol. Sci..

[66] Przybyłek I., Karpin´ski T.M. (2019). Antibacterial properties of propolis. Molecules.

